# Identifying the neural marker of chronic sciatica using multimodal neuroimaging and machine learning analyses

**DOI:** 10.3389/fnins.2022.1036487

**Published:** 2022-11-30

**Authors:** Xiaoya Wei, Liqiong Wang, Fangting Yu, Chihkai Lee, Ni Liu, Mengmeng Ren, Jianfeng Tu, Hang Zhou, Guangxia Shi, Xu Wang, Cun-Zhi Liu

**Affiliations:** ^1^International Acupuncture and Moxibustion Innovation Institute, School of Acupuncture- Moxibustion and Tuina, Beijing University of Chinese Medicine, Beijing, China; ^2^Department of Radiology, Beijing Hospital of Traditional Chinese Medicine Affiliated to Capital Medical University, Beijing, China; ^3^School of Life Sciences, Beijing University of Chinese Medicine, Beijing, China

**Keywords:** brain networks, chronic pain, fMRI, chronic sciatica, support vector machines, ALFF, cortical surface area

## Abstract

**Introduction:**

Sciatica is a pain disorder often caused by the herniated disk compressing the lumbosacral nerve roots. Neuroimaging studies have identified functional abnormalities in patients with chronic sciatica (CS). However, few studies have investigated the neural marker of CS using brain structure and the classification value of multidimensional neuroimaging features in CS patients is unclear.

**Methods:**

Here, structural and resting-state functional magnetic resonance imaging (fMRI) was acquired for 34 CS patients and 36 matched healthy controls (HCs). We analyzed cortical surface area, cortical thickness, amplitude of low-frequency fluctuation (ALFF), regional homogeneity (REHO), between-regions functional connectivity (FC), and assessed the correlation between neuroimaging measures and clinical scores. Finally, the multimodal neuroimaging features were used to differentiate the CS patients and HC individuals by support vector machine (SVM) algorithm.

**Results:**

Compared to HC, CS patients had a larger cortical surface area in the right banks of the superior temporal sulcus and rostral anterior cingulate; higher ALFF value in the left inferior frontal gyrus; enhanced FCs between somatomotor and ventral attention network. Three FCs values were associated with clinical pain scores. Furthermore, the three multimodal neuroimaging features with significant differences between groups and the SVM algorithm could classify CS patients and HC with an accuracy of 90.00%.

**Discussion:**

Together, our findings revealed extensive reorganization of local functional properties, surface area, and network metrics in CS patients. The success of patient identification highlights the potential of using artificial intelligence and multimodal neuroimaging markers in chronic pain research.

## Introduction

Sciatica is a pain disorder often caused by the herniated disk compressing the lumbosacral nerve roots, usually presenting as pain radiating from the low back down to the leg below the knee (Porchet et al., 2002; Deyo and Mirza, 2016). About a quarter of adults in the USA have experienced low back pain in the past 3 months, and 30% of those accompanied sciatica (Jensen et al., 2019). The global prevalence of sciatica varies from 1.2 to 43% (Konstantinou and Dunn, 2008; Finley et al., 2018), reflecting its ununified diagnostic criteria and diverse clinical manifestations. Pain caused by sciatica can easily progress to a chronic stage which may be either continuous or recurrent, and severely affects the quality of life and mental health (Foster and Reddington, 2021). However, the pathophysiologic mechanisms of chronic sciatica (CS) are not clear, which restricts the development of therapeutic protocols.

Previous neuroimaging studies have shown greater regional homogeneity (REHO) of the posterior cingulate (Liu et al., 2020) and lower functional connectivity (FC) between the dorsolateral prefrontal cortex (DLPFC) and anterior cingulate cortex (ACC) (Li et al., 2012) in patients with CS compared with healthy controls (HCs). However, these functional abnormalities may not fully account for the pathophysiology of CS, because a large number of studies have found both functional and structural (e.g., cortical surface area) changes associated with chronic pain (Seminowicz et al., 2011; Luchtmann et al., 2014; De Pauw et al., 2019; Niddam et al., 2019). Besides, multiple studies have suggested that communication between brain networks is changed in chronic pain patients, and connections across networks may reflect the presence of chronic pain (Kim et al., 2013; Hemington et al., 2016).

Given these neuroimaging findings on chronic pain, we speculated that patients with CS may also have abnormal changes in structural properties or between-regions FC. In addition, previous studies have applied machine learning techniques to distinguish patients with post-herpetic neuralgia (PHN) and HC using the amplitude of low-frequency fluctuation (ALFF) values (Huang et al., 2020). However, few studies have classified neuropathic pain patients from HC by multimodal neuroimaging features. The multidimensional neuroimaging features may serve as a bridge between clinical observations and neural mechanisms that can increase the understanding of CS as a complex and multifaceted pain-related disease.

Therefore, this study aimed to investigate the underlying neurobiological mechanisms of CS using surface-based morphometry, local functional metric, and network FC analyses in patients with CS using structural and functional magnetic resonance imaging (fMRI) data. Besides, the relationships between neuroimaging measures and clinical symptom scale scores were examined. Furthermore, the diagnosability of the neuroimaging properties was evaluated utilizing a support vector machine (SVM) of machine learning techniques and neuroimaging features with significant differences between CS patients and HC.

## Materials and methods

### Participants

This study included 34 CS patients who meet the diagnostic criteria of sciatica (Jensen et al., 2019) and 38 HC participants. Patients were recruited in the Dongzhimen Hospital Affiliated to Beijing University of Chinese Medicine from December 2020 to May 2021. The study recruited participants through hospital outpatient, the WeChat official account (one of China’s popular social media platforms) of Dongzhimen Hospital, and brochures.

The key inclusion criteria of CS people were: (1) 35–65 years old; (2) having unilateral radiating leg pain below the knee for more than 3 months, accompanied by a positive straight-leg raise test or corresponding neurological deficit (paresthesia, muscle weakness, or reflex abnormalities) with magnetic resonance imaging (MRI) or computed tomography (CT) confirmed disk herniation, (3) leg pain intensity on the visual analog scale (VAS) (0–100 mm) of 40 mm or higher (Collins et al., 1997), (4) right-handed. The exclusion criteria were: (1) sciatica induced by other diseases than lumbar disk herniation, (2) having the severe spinal disease or severe progressive neurological symptoms, (3) having cardiovascular, liver, kidney, or hematopoietic system diseases, mental health disorders, or other severe coexisting diseases, (4) pregnant or lactating women or those planning to conceive during the trial. Additionally, 38 pain-free age- and sex-matched HCs were recruited from the same geographic area by public advertisement. All HCs also met the above exclusion criteria. In addition, HCs were asked whether had personal or family histories of pain disorders or had experienced any significant pain condition as the exclusion criteria.

This study has been approved by the Ethics Committee of Dongzhimen Hospital Affiliated to Beijing University of Chinese Medicine (No. 2020BZYLL0803), and it was part of a study registered in Chinese Clinical Trial Registry (ChiCTR2100044585). All participants provided written informed consent according to the Declaration of Helsinki after study procedures were explained to them thoroughly. We collected MRI data from all participants.

### Clinical parameters

After recruitment, the following clinical measurements were evaluated by CS patients within the day before the MRI scanning. VAS was performed to rate the extent of pain in the leg and low back. Oswestry Disability Index (ODI) (Fairbank and Pynsent, 2000) was conducted to identify self-reported function levels through examining perceived disability in 10 activities of daily living. Sciatica Frequency and Bothersomeness Index (SFBI) (Atlas et al., 1996) was used to assess the frequency and bothersomeness of sciatica with scores ranging from 0 to 24, respectively. The 36-item Short-Form Health Survey (SF-36) (Lam et al., 2005) was administered to assess the quality of life in eight aspects, and the scores on the physical and mental components of the SF-36 will be summarized.

### Magnetic resonance imaging acquisition

Magnetic resonance imaging images were obtained at a Siemens 3.0 T MRI scanner (Skyra, Siemens, Erlangen, Germany) using a standard head coil at the Department of Radiology for Beijing Hospital of Traditional Chinese Medicine Affiliated to Capital Medical University. The high-resolution T1 structural MRI (sMRI) was acquired using a gradient echo sequence with the following parameters: repetition time (TR) = 2,530 ms, echo time (TE) = 2.98 ms, flip angle (FA) = 7°, inversion time = 1,100 ms, field of view (FOV) = 240 mm × 240 mm, number of slices = 192, voxel size = 1 mm × 1 mm × 1 mm, and in-plane resolution = 256 × 256. And resting-state functional MRI (rs-fMRI) was scanned using echo-planar imaging (EPI) sequence with the following parameters: whole brain, TR = 2,000 ms, TE = 30 ms, FOV = 224 mm × 224 mm, FA = 90°, slice thickness/gap = 3.5/0.6 mm, voxel size = 3.5 mm × 3.5 mm × 3.5 mm, axial slices = 32, in-plane resolution = 64 × 64, and 240 volumes. The scan duration was 5 min for the T1-weighted image and 8 min for EPI scans for blood oxygen-level dependent (BOLD)-based functional neuroimaging. We used comfortable foam pads to minimize head motion and earplugs to reduce noise interference. Before starting scanning, we instructed participants to keep their eyes closed, stay awake, avoid engaging in any specific thoughts and keep still.

### Quality control of magnetic resonance imaging data

Visually checking image quality by a neuroradiologist (QR) to make sure there were no apparent structural abnormalities or artifacts present, and the images with head movement greater than 2 mm in any direction or head rotation greater than 1° were excluded. Two HCs were excluded from the study on account of excessive head motion (>2 mm in translation or >2.0° in rotation) during the rs-fMRI scanning. The two excluded participants were female, their ages were 48 and 54 years. As a result, 34 patients with CS and 36 HCs were included in further statistical analyses. Furthermore, we also extracted the mean framewise displacement (FD) (Van Dijk et al., 2012) for each participant to measure the extent of head motion and compared them between the two groups. The Mann–Whitney *U* of non-parametric test result showed that there is no significant difference in head motion among the three groups (*z* = 1.575, *p* = 0.115).

### Structural magnetic resonance imaging data processing

First, the ‘‘recon-all’’ command with --all --qcache options implemented in FreeSurfer (V6.0)^1^ was used to pre-process T1-weighted images, the key steps including motion correction, non-uniform intensity normalization, talairach transform computation, skull removal, volumetric segmentation, cortical surface reconstruction and so on. Mean cortical thickness and surface area were calculated for each of the 68 cortical regions of the Desikan-Killiany Atlas (34 per hemisphere). Cortical thickness was estimated for each participant using the distance from the white matter boundary to the corresponding pial surface (Fischl and Dale, 2000). The cerebral surface area was calculated by mesh generation and surface triangulation. Then mean cortical thickness and surface area were extracted for each cortical region.

### Functional magnetic resonance imaging data processing and network analyses

The fMRI data were pre-processed using the software MATLAB 2017 and the toolbox for Data Processing and Analysis for Brain Imaging (DPABI) (version 6.1)^2^ (Yan et al., 2016). For each participant’s image data, we discarded the first 10 volumes because of signal equilibrium, a total of 230 volumes for each subject were processed with the slice timing, motion correction, spatial smoothing (8-mm FWHM), and spatial normalization to the Montreal Neurological Institute (MNI) space. Then we re-sampled the data into 3 mm × 3 mm × 3 mm. Finally, after removing the linear trend, we applied a 0.01–0.08 Hz bandpass filter.

It should be noted that ALFF was calculated without filtering during the pre-processing process, and REHO was not smoothed during the pre-processing but smoothed after it was calculated, to allow the data to be normalized, which would be conducive to statistical analysis and indicator standardization. ALFF and REHO values were calculated using the DPBAI toolbox. ALFF is used to detect the regional intensity of spontaneous fluctuations in the BOLD signal, REHO calculates the temporal homogeneity of the BOLD signal between a given voxel with neighboring voxels. These measures were selected to pinpoint the spontaneous neural activity of specific regions and physiological states of the brain. The ALFF measures the gross power of oscillations within a certain frequency range, using the DPBAI software and regions of interest (ROIs) defined by the Anatomical Automatic Labeling (AAL) ROI library. The calculation procedure: (1) Fast Fourier Transform (FFT) was used to convert all voxels from the time domain to the frequency domain; (2) the ALFF of every voxel was calculated by averaging the square root of the power spectrum across 0. REHO was computed based on Kendall’s coefficient of concordance (KCC) of the time series of the voxel with its nearest 26 neighboring voxels. The REHO was computed for all brain voxels.

We used the software MATLAB 2017 and the DPABI (version 6.1) to extract time courses of 160 ROIs in the Dosenbach 160 atlas (Dosenbach et al., 2010). Each ROI (i.e., node) was a 5 mm radius sphere centered on the atlas coordinates, including 19 voxels in each. To derive the connectivity matrix of the brain, we computed Pearson correlation coefficients of BOLD signals between each pair of 142 ROIs (Glasser et al., 2016) (Dosenbach 160 atlas exclude 18 ROIs of the cerebellum), which were then Fisher transformed to *z*-values. We grouped significant nodes according to a well-defined seven-network atlas derived from 1,000 healthy participants by Yeo et al. (2011): sensory-motor network (SMN), ventral attention network (VAN), visual network (VN), dorsal attention network (DAN), default mode network (DMN), frontoparietal network (FPN), and subcortical network (SC). Because the limbic network nodes from the Yeo atlas were not covered by the Dosenbach 160 atlas, we defined subcortical ROIs as the SC (Yang et al., 2021).

### Statistical analyses

#### Demographic and clinical characteristics analyses

Demographic data collected from either group includes age, gender, educational level, and occupation. Participants were asked to indicate the physical activity level of the work they do most of the time, the nature of occupation was defined as manual work and mental work. We used Statistical Package for Social Sciences (SPSS) V21 software to conduct statistical analyses. Before statistical analyses, we checked the normality of each metric. Education in each group and age of the HC group were non-normally distributed, we used Kolmogorov–Smirnov non-parametric tests. As for categorical variables (i.e., gender and occupation), we used the Chi-Square test to evaluate the differences between groups. The significance level was set at *p* < 0.05.

#### Surface area and thickness analyses

The cortical surface area and cortical thickness of CS patients and HC were extracted. Then, we used SPSS V21 software to conduct statistical analyses. Two-sample independent *t*-tests were used to compare the regional-wise differences between the two groups if the measurements were normally distributed [False discovery rate (FDR) correction, *p* < 0.05], and if the data distribution is not normal, we used non-parametric tests of Mann–Whitney *U*. Effect sizes are depicted as Cohen’s d. The effect size was computed at https://www.psychometrica.de/effect_size.html.

#### Amplitude of low-frequency fluctuation and regional homogeneity analyses

For ALFF and REHO maps, voxel-wise two-sample independent *t*-tests were performed to compare the results between the two groups, Gaussian Random Field theory (GRF) correction, voxel-level *p* < 0.001, and cluster-level *p* < 0.05. We extracted the values of ALFF and REHO results and calculated effect sizes using Cohen’s d.

#### Network functional connectivity analyses

For FC analyses, we also used two-sample independent *t*-tests with FDR corrected (*p* < 0.05) in DPABINet (See text footnote 2, version 1.1). The figures were distributed in DPABINet and BrainNet Viewer.^3^ Finally, we extracted the values and showed them in the tables. Cohen’ s d was used as the effect size measure.

#### Brain metrics and clinical variables correlation analyses

We extracted metrics (ALFF, REHO, surface-based morphometry, and FC) with significant group differences and investigated their relationships with clinical variables. VAS score for leg pain, VAS score for back pain, ODI score, SF-36 for physical, SF-36 for mental, SFBI for frequency, and SFBI for bothersomeness were investigated. For non-normally distributed variables (VAS score for leg pain and SF-36 for mental score), we used Spearman’s correlation analyses. For the other normally distributed variables, Pearson correlation was used to analyze the correlation. The above statistical analyses were implemented using SPSS V21 (significance level is *p* < 0.05).

#### Group classification with support vector machine

After revealing the significant ALFF values, surface area, and FCs in the CS group, we used these three kinds of features to accurately differentiate the 34 CS individuals from the 36 HCs. Features with different scales across different modalities were normalized to a value between 0 and 1 according to their maximum and minimum values. Then, the discriminant analysis was performed by using the SVM with a nested leave-one-out cross-validation (LOOCV) framework. First, the C regularization parameter and the linear kernel function were optimized by performing 5-fold cross-validation on the n-1 (i.e., 69) training data. Once the optimal SVM model was obtained, it was applied to classify the left-out individual as CS or HC.

The performances of a classifier were quantified using accuracy, sensitivity, specificity, and the area under the receiver operating characteristic (ROC) curve (AUC). Note that the specificity represented the proportion of the HC individuals correctly predicted, while the sensitivity represented the proportion of the CS individuals correctly predicted. Specifically, accuracy is calculated as (TP + TN)/(TP + TN + FN + FP), sensitivity is defined as TP/(TP + FN) and specificity is defined as TN/(FP + TN), where TN is the number of true negatives (HC individuals correctly classified), TP is the number of true positives (CS individuals correctly classified), FN is the number of false negatives (CS individuals classified as HC individuals), and FP is the number of false positives (HC individuals classified as CS individuals). In addition, the AUC is an evaluation measure based on the ROC curve, which illustrates the performance of the classifier. The ROC curve is delineated by plotting 1-specificity and sensitivity at different thresholds, and the thresholds of each ROC curve underwent stepwise variation from 0 to 1 in each 0.1 interval. Last, the model’s performance was evaluated by computation of the Matthews Correlation Coefficient (MCC). The calculation formula (Ali et al., 2021) is as follows:


$$MCC=\frac{TP\times TN-FP\times FN}{\surd \left(TP+FP\right)\left(TP+FN\right)\left(TN+FP\right)\left(TN+FN\right)}$$

## Results

### Demographic and clinical characteristics

Thirty-four CS patients and 36 matched HCs completed the entire study. Age and years of education were not normally distributed, so the Kolmogorov–Smirnov test was used to test for group differences. No significant group differences were found in age (*p* = 0.449), gender (Chi-square test: *p* = 0.497), years of education (*p* = 0.381), and occupation (Chi-square test: *p* = 0.204) between the CS and HC groups (Table 1). And the median pain duration of CS was 8 months, the median pain score for the leg on the VAS was 55, and the mean VAS score for back pain was 57.50 (14.72) in patients. Otherwise, the mean SF-36 score for the physical duration of CS was 35.32 (10.34), and the median SF-36 score for mental was 57.31 in CS patients.

**TABLE 1:** Demographic and clinical characteristics of two groups.

Parameter	CS (*n* = 34)	HC (*n* = 36)	Statistics	*P*-value
Age (years)	54.29 (8.80)	58.50 (51, 62.75)	*Z* = 0.861	0.449^a^
Gender (M/F)	14/20	12/24	*X*^2^ = 0.461	0.497^b^
Education (years)	12.97 (3.49)	11.50 (2.77)	*Z* = 0.909	0.381^a^
Occupation (Men/Man)	11/23	19/17	*X*^2^ = 1.611	0.204^b^
Pain duration (years)	8.00 (3.38, 17.75)	N/A	N/A	N/A
VAS score for leg pain	55 (50, 70)	N/A	N/A	N/A
VAS score for back pain	57.50 (14.72)	N/A	N/A	N/A
ODI score	26.27 (11.33)	N/A	N/A	N/A
SF-36 for physical	35.32 (10.34)	N/A	N/A	N/A
SF-36 for mental	57.31 (47.42, 62.71)	N/A	N/A	N/A
SFBI for frequency	12.85 (4.84)	N/A	N/A	N/A
SFBI for bothersomeness	11.88 (4.27)	N/A	N/A	N/A

We used mean (standard deviation) if the measurements were normally distributed, and median (Q1, Q3) if the measurements were not normally distributed. ^a^Non-parametric test, Kolmogorov–Smirnov. ^b^Chi-square test. CS, chronic sciatica; HC, healthy controls; M/F, male/female; Men/Man, Mental work/Manual work; N/A, not applicable; VAS, visual analog scale, 0–100 mm; ODI, Oswestry Disability Index; SF-36, the 36-item Short-Form Health Survey; SFBI, Sciatica Frequency and Bothersomeness Index.

### Amplitude of low-frequency fluctuation abnormality in chronic sciatica patients

**FIGURE 1:**
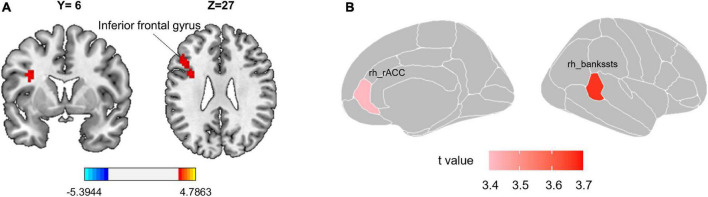
Group differences in local functional metric and surface morphology. **(A)** Patients with CS have significantly higher ALFF value in the left inferior frontal gyrus. The red color reflects ALFF values greater in CS patients than in the HC group. Gaussian Random Field theory (GRF) correction with voxel-level *p* < 0.001 and cluster-level *p* < 0.05. **(B)** Cortical surface area differences between CS patients and HC. *T*-values of two altered brain regions in the surface area of the right hemisphere. Positive (red) values reflect cortical area larger in CS patients than in the HC group, FDR corrected *p* < 0.05. CS, chronic sciatica; CS, chronic sciatica; HC, healthy controls; rh, right hemisphere; rACC, rostral anterior cingulate cortex; bankssts, banks of superior temporal sulcus.

Compared with the HC group, patients with CS had higher ALFF in the left inferior frontal gyrus (IFG) (*t* = 4.132, ES = −1.238, CI [−1.750 to −0.727]; Figure 1A and Table 2). However, the REHO analysis did not yield any significant results at the whole brain level.

**TABLE 2:** Significant differences in ALFF between two groups.

	Regions	Peak MNI coordinates			Voxels size	*t*-value	ES (95%CI)
		*X*	*Y*	*Z*			
**ALFF**							
CS > HC	Inferior frontal gyrus, L	−36	6	27	85	4.132	−1.238 (−1.750, −0.727)

Regions were identified in Figure 1A. Peak coordinates (*X*, *Y*, *Z*) are displayed according to MNI standard space, and labels according to the AAL atlas. GRF corrected, voxel-level *p* < 0.001, cluster-level *p* < 0.05. MNI, Montreal Neurological Institute; CS, chronic sciatica; HC, healthy controls; ALFF, the amplitude of low-frequency fluctuation; L, left; ES, effect sizes, Cohen’s d; CI, confidence interval; the effect size was computed for groups with different sample size.

### Abnormal surface area in chronic sciatica patients

Compared with the HC group, patients with CS had the larger surface area in the right banks of the superior temporal sulcus (bankssts) (*t* = 3.666, ES = −0.877, CI [−1.367 to 0.386], FDR corrected *p* = 0.016) and right rostral anterior cingulate (rACC) (*t* = 3.417, ES = −0.817, CI [−1.305 to 0.329], FDR corrected *p* = 0.018; Table 3 and Figure 1B). However, we did not find a significant difference in cortical thickness between the two groups.

**TABLE 3:** Differences in surface area index between the two groups.

	Region	CS (*n* = 34)	HC (*n* = 36)	*t*-value	ES (95%CI)	*P*-value
CS > HC	rh_bankssts	941.24 (150.03)	834.78 (86.14)	3.666	−0.877 (−1.367, 0.386)	0.016*
	rh_rACC	662.29 (170.19)	542.17 (121.17)	3.417	−0.817 (−1.305, 0.329)	0.018*

We used mean (standard deviation) if the measurements were normally distributed. Two-sample *t*-test. *Survives false discovery rate (FDR) correction, *p* < 0.05. CS, chronic sciatica; HC, healthy controls; rh, right hemisphere; rACC, rostral anterior cingulate cortex; bankssts, banks of superior temporal sulcus; ES, effect sizes, Cohen’s d; CI, confidence interval; the effect size was computed for groups with different sample size.

### Functional connectivity alterations in chronic sciatica and its relationship with clinical symptoms

**FIGURE 2:**
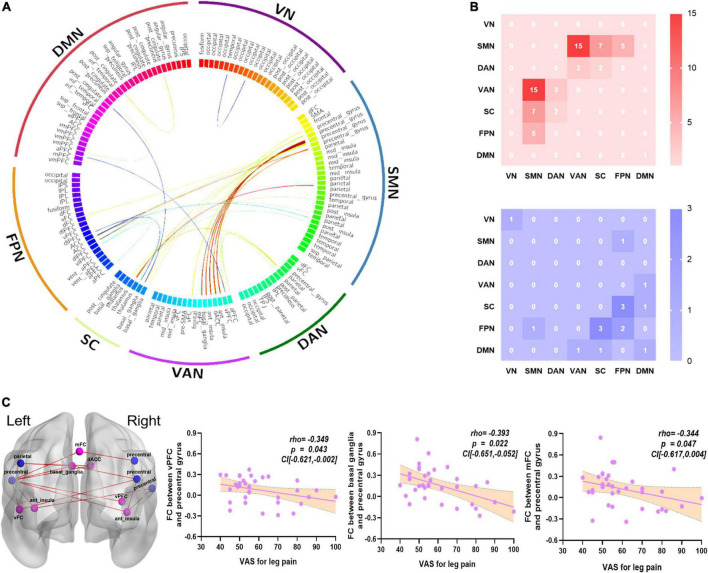
The altered FCs between networks and relationships with clinical symptoms. **(A)** The FCs significantly altered in the CS group compared with the HC group, which were mainly focused on SMN and VAN (*p* < 0.05, FDR corrected). **(B)** 15 greater FCs were involved in the SMN and VAN. The red color represents greater FCs; the blue color represents lower FCs. **(C)** The VAS for leg pain score had a negative correlation with the FC of right vPFC and left precentral gyrus (Spearman, *r* = –0.349, *p* = 0.043, CI [–0.621 to –0.002]); the FC of left basal ganglia and left precentral gyrus (Spearman, *r* = –0.393, *p* = 0.022, CI [–0.651 to –0.052]); and the FC of mFC and left precentral gyrus (Spearman, *r* = –0.344, *p* = 0.047, CI [–0.617 to 0.004]). The blue color nodes belong to SMN, the light purple nodes belong to VAN, the red color represents greater FCs. CS, chronic sciatica; HC, health control; vPFC, ventral prefrontal cortex; mFC, medial frontal cortex; SMN, somatomotor network; VAN, ventral attention network; VN, visual network; DAN, dorsal attention network; DMN, default mode network; FPN, frontoparietal network; SC, subcortical network; VAS, visual analog scale.

For FC analysis, there were 15 connections between SMN and VAN that exhibited higher connection strength in the CS group than in the HC group, with a few connections among other networks. In addition, there were 10 lower connections among six networks (*p* < 0.05, FDR corrected, Table 4 and Figures 2A,B). In addition, the FC between right vPFC and left precentral gyrus had a negative correlation with the VAS for leg pain score (Spearman *rho* = −0.349, *p* = 0.043, CI [−0.621 to −0.002]), the FC between left basal ganglia and left precentral gyrus had a negative correlation with the VAS for leg pain score (Spearman *rho* = −0.393, *p* = 0.022, CI [−0.651 to −0.052]), and the FC between mFC and left precentral gyrus had a negative correlation with the VAS for leg pain score (Spearman *rho* = −0.344, *p* = 0.047, CI [−0.617 to 0.004]). The correlation results were shown in Figure 2C.

**TABLE 4:** The comparison of FCs between two groups.

Comparisons	Significant FC		Group		*t*-value	ES (95%CI)	*P*-value
	Region A	Region B	CS (*n* = 34)	HC (*n* = 36)			
Sciatica > HC	vPFC	Precentral	0.08 (0.18)	0.09 (0.06)	4.171	0.106 (−0.363, 0.575)	<0.05*
	Ant insula	Precentral	0.13 (0.14)	−0.11 (0.18)	4.047	−1.409 (−1.932, −0.885)	<0.05*
	Ant insula	Precentral	0.07 (0.18)	−0.05 (0.20)	4.356	−0.599 (−1.079, −0.120)	<0.05*
	Ant insula	Precentral	0.08 (0.19)	−0.12 (0.19)	4.848	−1.079 (−1.580, −0.577)	<0.05*
	dACC	Precentral	0.12 (0.25)	−0.13 (0.17)	5.062	−1.145 (−1.650, −0.639)	<0.05*
	dACC	Parietal	0.08 (0.25)	−0.17 (0.23)	4.530	−1.064 (−1.564, −0.563)	<0.05*
	Ant insula	Precentral	0.16 (0.19)	−0.18 (0.23)	4.258	−1.618 (−2.158, −1.078)	<0.05*
	Ant insula	Precentral	0.13 (0.17)	−0.04 (0.21)	5.014	−0.920 (−1.413, −0.427)	<0.05*
	Basal ganglia	Precentral	0.17 (0.21)	−0.08 (0.19)	3.913	−1.294 (−1.810, −0.779)	<0.05*
	Basal ganglia	Precentral	0.16 (0.24)	−0.04 (0.25)	4.869	−0.827 (−1.316, −0.339)	<0.05*
	Basal ganglia	Parietal	0.12 (0.23)	−0.11 (0.22)	4.538	−1.006 (−1.504, −0.509)	<0.05*
	mFC	Precentral	0.12 (0.25)	−0.13 (0.23)	5.142	−1.040 (−1.540, −0.541)	<0.05*
	mFC	Precentral	0.18 (0.23)	−0.15 (0.19)	3.981	−1.599 (−2.137, −1.061)	<0.05*
	vFC	Precentral	0.17 (0.21)	−0.03 (0.21)	4.094	−0.958 (−1.453, −0.463)	<0.05*
	vFC	Precentral	0.26 (0.20)	0.0001 (0.14)	4.324	−1.511 (−2.042, −0.980)	<0.05*

We used mean (standard deviation) if the measurements were normally distributed. CS, chronic sciatica; HC, health control; vPFC, ventral prefrontal cortex; ant insula, anterior insula; dACC, dorsal anterior cingulate cortex; mFC, medial frontal cortex; vFC, ventral frontal cortex. *Survives false discovery rate (FDR) correction, *p* < 0.05. ES, effect sizes, Cohen’s d; CI, confidence interval; the effect size was computed for groups with different sample size.

### Multimodal brain metrics discriminate between chronic sciatica patients and healthy control

**FIGURE 3:**
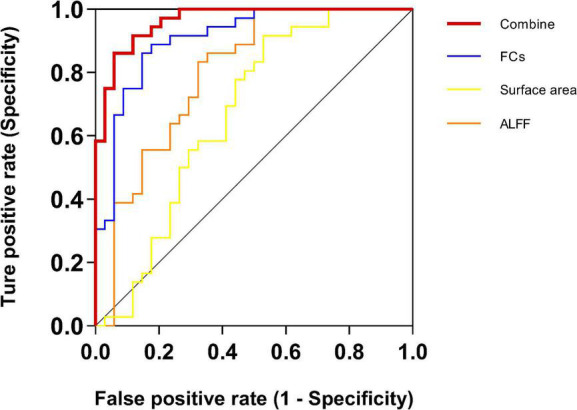
Receiver operating characteristic (ROC) for CS and HC SVM classification analyses. For single-measurement analyses, the functional connectivity (FC) exhibited a higher AUC of 90.85% than the other two measurements. The surface area achieved the lowest AUC of 66.99%. Critically, the AUC improved after combining all features of the three measurements, achieving an AUC up to 96.41%. ALFF, the amplitude of low-frequency fluctuation; FC, functional connectivity.

In this study, ALFF, surface area, and FCs were utilized to classify whether a sample belonged to the CS group (Table 5 and Figure 3). For single-measurement analyses, the FCs exhibited a higher accuracy rate (accuracy = 85.71%) and MCC of 0.715 than the ALFF (accuracy = 70.00%, and MCC = 0.424) and surface area (accuracy = 68.57%, MCC = 0.398). Surface morphology achieved the lowest accuracy rate. The classification accuracy improved after combining the significant measurements of the three features, achieving an accuracy of 90.00%, an AUC of 0.96, and an MCC of 0.800.

**TABLE 5:** Results of the discrimination analyses derived from the SVM between HC and CS.

Feature	Accuracy (%)	Sensitivity (%)	Specificity (%)	AUC (%)	MCC
ALFF	70.00	67.65	83.33	79.66	0.424
Surface area	68.57	55.88	77.78	66.99	0.398
FCs	85.71	85.29	86.11	90.85	0.715
Combining features	90.00	94.12	86.11	96.41	0.800

For single-measurement analyses, the FCs exhibited a higher accuracy rate (85.71%) than the other two features. The surface morphology (surface area) achieved the lowest accuracy rate of 68.57%. The classification accuracy improved after combining the features of the three measurements, achieving an accuracy of up to 90.00%. CS, chronic sciatica; HC, health control; ALFF, the amplitude of low-frequency fluctuation; FC, functional connectivity, AUC, the area under the curve; MCC, Matthews Correlation Coefficient; FC, functional connectivity; SVM, support vector machine.

## Discussion

Combining a variety of analysis methods, we demonstrated that CS patients had abnormal local neural activity, which was also reflected in the greater ALFF values of the left IFG. At a finer cortical scale, we could identify the significantly greater cortical surface area in the regions cingulate and temporal. At the resting-state functional network level, we found that CS patients showed greater FCs mainly between the SMN and VAN, especially the precentral gyrus and anterior insula. Finally, we found that multimodal combined neuroimaging features were more dominant in this disease classification performance.

### Greater surface area of rostral anterior cingulate and banks of the superior temporal sulcus in right hemisphere

The ACC plays a vital role in the neuropathic pain effect in animals (Gao et al., 2020). For instance, increased GABAergic inhibitory control in the rACC could reduce ongoing pain and pain aversiveness caused by sciatic nerve injury (Juarez-Salinas et al., 2019). Using fMRI and electrophysiological recording, a previous study observed plasticity changes in the cingulate cortex in rats with neuropathic pain (Chao et al., 2018). However, the underlying role of the ACC in CS patients is much unclear. Our study showed a larger surface area in the rACC in CS patients compared to HC, suggesting that the rACC may be related to pain-induced negative emotion in CS patients. In another neuropathic pain disease, trigeminal neuralgia patients exhibited reduced ACC surface area compared with HC (Mo et al., 2021). The controversy may stem from different neuropathological processes. In the future, we will further explore whether the enlarged surface area of ACC can be the biomarker to distinguish CS patients from other neuropathic pain disorders.

Furthermore, the superior temporal gyrus might be involved in pain due to mismatches between pain expectation and perception (Smallwood et al., 2013). Patients with chronic traumatic neck pain showed a smaller cortical volume in the right superior temporal gyrus compared to HC (De Pauw et al., 2019). However, our study showed that CS patients have a greater cortical surface area in the right superior temporal gyrus, which may be due to the different etiologies and neuropathological processes of diseases. A study reported that patients with bipolar disorder showed a larger surface area of left bankssts, which could help distinguish them from patients with major depression, the overall accuracy was 74.3% (Fung et al., 2015). The surface area of the right bankssts and rACC in this study were also effective in distinguishing CS patients from HC, the accuracy was 66.99%. These results suggested that disease-specific neuroanatomical features (e.g., cortical surface area) may help establish reliable distinctions between different populations (e.g., between different types of disease, between patients with HC).

### Frontal cortex showed greater spontaneous neuronal activity and functional connectivity’s

In our study, we found significantly greater ALFF in the IFG in CS patients compared to HC. Consistent with previous studies involving aspects of chronic pain (Buckalew et al., 2010; Hashmi et al., 2013), we found the neural activity of the frontal lobe was significantly greater in CS patients. IFG is the important part of the prefrontal cortex (PFC), the brain region commonly associated with cognitive and emotional processing (Petrides, 2005; He et al., 2007). In CS patients, the abnormal functional activity of the two brain regions may influence pain perception through heightening emotional responses to pain (Gracely et al., 2004).

Consistent with a previous study, medial PFC/rACC had abnormally increased FCs with brain regions with the SMN (postcentral gyrus) in cLBP patients, and the FCs could discriminate cLBP patients from HCs with 91% accuracy (Tu et al., 2020a). Our study found the greater FCs between the vPFC, mFC, and the precentral gyrus (SMN), indicating that communication between the frontal cortex and sensory-related regions was altered in CS patients. In addition, the two FCs were negatively correlated with the pain intensity of the leg, suggesting that the feeling of pain caused by CS is the main symptom and significantly reduces the patient’s quality of life. However, DMN connectivities in the patients with cLBP and/or pain in a lower vs. the HC showed reductions of this network in the dorsolateral PFC, medial PFC, and ACC (Li et al., 2014). It may be due to its small sample size (20 patients and 10 HCs) and only focus on FCs within the DMN. Based on these results, we speculated that the persistent chronic pain and associated symptoms of CS were caused by abnormalities in frontal cortex.

### The important functional connectivity’s were mainly between sensory-motor network and ventral attention network regions

The SMN including the primary and secondary sensorimotor cortex, which receives and processes sensory information from the periphery, is thought to be the main brain network responsible for pain perception (Mayer et al., 2015). Abnormalities in the VAN were also widely seen in chronic pain patients with persistent diminished attention or inattention (Moriarty et al., 2011; Wen et al., 2012). The pathological changes of basal ganglia (Borsook et al., 2010; Starr et al., 2011) and neurological dysfunction of the anterior insular cortex (Ferraro et al., 2022) in the VAN have also been reported to be involved in pain processing leading to altered pain perception. Patients with failed back surgery syndrome with chronic low back pain have greater FC in the precentral gyrus and putamen (extending to the insula) in the SMN compared to HC (Kolesar et al., 2017). Consistent with previous studies, the FC between SMN and VAN was higher in CS patients, especially between the precentral gyrus and anterior insula, compared with HC. In addition, patients with CLBP had greater gray matter volume in the SMN regions and greater FC between the bilateral sensorimotor cortex and sensory association cortex during pain (Li et al., 2018). These studies may imply that enhanced cortical activity in the SMN and VAN regions also underlies the clinical pain status of CS patients. Furthermore, the precentral gyrus is a sensorimotor area that receives information projected from the basal ganglia, both of which play an important role in pain processing (Liu et al., 2012). Compared to HC, complex regional pain syndrome patients displayed greater resting connectivity from the caudate to the primary motor cortex (Lee et al., 2022). Our study found a greater FC between the basal ganglia and the precentral gyrus in CS patients, indicating that pain may increase the attention of CS patients to their pain sensation, and the FC was negatively correlated with the VAS score of leg pain, we speculate that engaging the conscious attention (Aminabadi et al., 2022) of CS patients can reduce pain perception.

Our study also showed the abnormal FCs within FPN and with the other networks, and previous studies have shown positively associated with pain rating changes (Kong et al., 2013). The frontoparietal region may play a dominant role in the formation and transmission of sensations (Lobanov et al., 2013), and RFPN is recognized as an important network that associates with perception and pain (Smith et al., 2009). Neural function activity in encephalic regions of FPN showed abnormal changes (Cui et al., 2022), and a significantly lower FC of RFPN was observed in MWoA patients (Li et al., 2015). In our study, we found increased FCs between FPN and SMN, and decreased FCs within FPN and with the other networks in CS patients, suggesting that FPN also plays an important role in the processing and regulation of CS pain.

### Multimodal metrics successfully distinguish chronic sciatica patients from healthy control

In recent years, SVM techniques combined with neuroimaging metrics have been applied to differentiate pain patients from HCs and to predict the outcome of certain interventions (Bagarinao et al., 2014; Zeng et al., 2019; Huang et al., 2020; Tu et al., 2020b; Gui et al., 2021; Wei et al., 2022). The patients with neuropathic pain and the HC were classified by the mean ALFF values of the frontal gyrus and the precuneus using the linear SVM classifier, and the classification accuracy was 86.36% between the PHN patients and HC (Huang et al., 2020). A study identified a neural marker with abnormal FC within the SMN and FPN that could discriminate MwoA patients from HC with a 91.4% accuracy rate (Tu et al., 2020b). Patients with trigeminal neuralgia exhibited reductions in cortical indices in the cingulate cortex, and these abnormal whole brain-level morphological alterations successfully enable automated trigeminal neuralgia diagnosis with high specificity (trigeminal neuralgia: 95.35%; disease controls: 46.51%) (Mo et al., 2021). Interestingly, these studies reported that the classification performance of FCs between networks was higher than the ALFF values and cortical indices (structural features). Despite coming from various studies about different chronic and neuropathic pain diseases, these studies showed that ALFF values, inter-network FCs, and structural measurements (e.g., cortical indices) can be used as neurological features to distinguish chronic pain patients from healthy people, respectively.

However, chronic pain could affect multiple brain systems and cause extensive reorganization of brain structure and function, and the results of these studies were often derived from a few modalities, ignoring the combination of multiple modalities, which may affect the performance of the machine learning classifiers. In our study, SVM was applied to combined MRI imaging features (ALFF, cortical surface area, FCs), which distinguished CS patients from HC with higher accuracy of 90.00%. The finding implies that multimodal data analysis gives better results and exhibits the best model’s performance (MCC = 0.800) than unimodal analysis. The multimodal analysis could combine the advantages of multiple imaging techniques to improve both spatial and temporal resolution and target disease neurobiomarkers with high specificity and sensitivity and provide many new opportunities to improve brain research. The identification of distinguishable or predictive neuroimaging biomarkers is needed as it can aid in diagnosis and prognosis, as well as be helpful in clinical decision-making. To date, one of the few factors that independently predict poor outcomes in sciatica is the duration of leg pain (Konstantinou et al., 2018), the application of multimodal neuroimaging biomarkers may help to assess the disease severity of patients and progression, especially during non-painful periods, assisting clinicians in early decision making, and tailor treatment plans for patients. For instance, they could be a useful diagnostic tool when patients are unable to communicate or self-reports are otherwise unreliable. Moreover, our findings may invite future studies with larger datasets to investigate the relationship between multimodal neuroimaging biomarkers and clinical measurements and develop therapeutic biomarkers to evaluate or predict the response of potential new treatments.

### Study limitations

We acknowledge that our research has several limitations. First, the present study was based on 34 CS patients and 36 HCs, it is necessary to expand the sample size to confirm the results. Brain metrics and clinical variables correlation analysis were conducted with two-sided significance levels (alpha = 0.05) without corrections for multiple comparisons due to the small sample size and the exploratory nature of the study. Second, the study covered a range of ages from 35 to 65 years, restricting the generalization of the present results to other populations. Future studies with the younger or older age range are needed to increase external validity. Third, it might be a lack of a dataset of patients with other chronic pain disorders, we could not verify the specificity of the multimodal markers. Future studies with more datasets of pain-related disease will help clarify which specific chronic pain diseases (e.g., knee osteoarthritis, low back pain) are associated with functional impairment in different brain domains. Fourth, perhaps even more importantly, clinical measurements were evaluated at a single time point (1 day before MRI scanning) which does not necessarily reflect the long-term status of an individual, other time-dependent variables on multiple time points may provide more information on the chronic pain status. Fifth, the results of the brain’s functional network are affected by different parcelation strategies. Other brain atlases are needed to further assess the reliability of the differentiation of CS individuals. Last, we acknowledged that we only recruited sciatica patients with diagnosed disk herniated disks, and it cannot distinguish if the results are due to pain, or due to the specific nature of the origins of the pain in the patient group, so our results should not be exaggerated.

## Conclusion

Our findings provide new insights into the pathophysiologic mechanisms of CS and highlight the potential of multimodal features as markers in the research of neural mechanisms of chronic pain.

## Data availability statement

The raw data supporting the conclusions of this article will be made available by the authors, without undue reservation.

## Ethics statement

The studies involving human participants were reviewed and approved by Ethics Committee of Dongzhimen Hospital Affiliated to Beijing University of Chinese Medicine (No. 2020BZYLL0803). The patients/participants provided their written informed consent to participate in this study.

## Author contributions

XYW: formal analysis, visualization, and writing – original draft and review and editing. XW: conceptualization, methodology, supervision, visualization, and writing – review and editing. GS: supervision, writing – review and editing, and investigation. LW and JT: supervision and funding acquisition. FY: data curation. CL, MR, and HZ: data curation and investigation. NL: supervision, data curation, and investigation. C-ZL: conceptualization, funding acquisition, and supervision. All authors contributed to the article and approved the submitted version.

## Footnotes

1
http://surfer.nmr.mgh.harvard.edu/

2
http://rfmri.org/dpabi

3
http://www.nitrc.org/projects/bnv
